# Effects of oxytocin and anaesthesia on vascular tone in pregnant women: a randomised double-blind placebo-controlled study using non-invasive pulse wave analysis

**DOI:** 10.1186/s12884-018-2029-1

**Published:** 2018-11-22

**Authors:** Sofus Rabow, Ull Hjorth, Sofia Schönbeck, Per Olofsson

**Affiliations:** 1 Department of Clinical Sciences Lund, Anaesthesiology and Intensive Care, Skåne University Hospital, Lund University, S-22185 Lund, Sweden; 2 Department of Clinical Sciences Malmö, Obstetrics and Gynaecology, Skåne University Hospital, Lund University, Malmö, Sweden; 3 Department. of Intensive and Perioperative Care, Skåne University Hospital, S-22185 Lund, Sweden

**Keywords:** Anaesthesia, Arterial stiffness, Oxytocin, Photoplethysmography, Placebo, Pregnancy, Pulse wave analysis, RCT, Vascular tone

## Abstract

**Background:**

Oxytocin is an uterotonic drug with profound cardiovascular effects, which in compromised patients could lead to serious events. The objective was to investigate whether oxytocin affects cardiac function and vascular tone in large and small arteries. We hypothesized that oxytocin decreases arterial vascular tone and elevates cardiac output.

**Methods:**

51 pregnant women were randomised to treatment with 8.3 μg (5 U) oxytocin or placebo injection during first trimester surgical evacuation of the gravid uterus under general anaesthesia. Oxytocin or placebo was administered once either early or late in the procedure, in a double-blind fashion. Digital photoplethysmography pulse wave analysis variables, heart rate, mean arterial blood pressure and electrocardiographic ST index were recorded before and after anaesthesia and after each injection. Non-parametric statistics were used with a two-sided *P* value < 0.05 considered significant.

**Results:**

Anaesthesia induced a significant fall in blood pressure, heart rate and vascular tone in small and peripheral arteries. Oxytocin had a vasodilatory effect on small and peripheral arteries and increased the left cardiac ventricular ejection time. The ST index decreased.

**Conclusions:**

Pulse wave analysis indicated peripheral vasodilation and increased cardiac output after oxytocin, implying increased myocardial oxygen demand. These effects might have been enhanced by the vasodilating effects of anaesthesia. Previous studies have demonstrated myocardial ischaemia after oxytocin, as reflected by a decrease in ST index in the present study.

**Trial registration:**

Trial registration number ISRCTN17860978, 2018/03/14, Retrospectively registered.

## Background

Oxytocin is used to contract the uterus for prevention and treatment of post-partum haemorrhage after both vaginal delivery and Caesarean section (CS) [1]. It is also used after curettage to prevent post-operative uterine atony and bleeding [2]. Oxytocin has cardiovascular side effects such as tachycardia, vasodilation, hypotension and chest pain. This may lead to serious cardiovascular events in an already compromised patient [3].

We have previously studied the cardiovascular effects of oxytocin using digital photoplethysmographic pulse wave analysis (DPA) during elective CS [4]. We found that oxytocin causes arterial vasodilation of both small and large arteries and has a direct negative chronotropic effect with increased left ventricular (LV) ejection power accompanied by electrocardiographic (ECG) ST changes. However, the combination of CS, spinal anaesthesia, intravenous fluids, vasoactive drugs and delivery of the baby and placenta, made it problematic to study the haemodynamic effects of oxytocin exclusively. Therefore, we wanted to conduct a study in a more stable clinical setting. For this purpose, we chose to study the circulatory effects in first trimester pregnant women undergoing uterine evacuation after miscarriage or for surgical termination of pregnancy.

The DPA method can assess cardiac LV ejection time (LVET) and distinguish between tonus changes in large and small arteries [5]. Using DPA in combination with ECG ST index and mean arterial blood pressure (MAP) measurements adds information whether there are direct cardiac effects of oxytocin [5, 6]. The method has been validated against invasive aortic pressure measurements during vasoconstrictory and vasodilatory manipulations [7][Takazawa 1998], the physiological background to the digital photoplethysmographic volume pulse wave contour characteristics has been thoroughly described [8, 9] and the method correlates well with the gold standard applanation tonometry method [5, 8]. The DPA has the advantage of being rapid, non-invasive, pain-free, and user independent.

## Methods

The primary objective of the study was to investigate whether oxytocin affects both large and small arteries, and cardiac LV ejection function, using the DPA method. Based on our own observations [4] and observations by Weis et al. [10], we hypothesised that oxytocin decreases arterial vascular tone and elevates cardiac output (CO). The regional research ethics committee in Lund approved the study (dnr 2012/649) and all enrolled women gave their informed oral and written consent. The study was performed in accordance with The Code of Ethics of the Declaration of Helsinki. The women were recruited consecutively at the Department of Obstetrics and Gynaecology at Skåne University Hospital, Malmö, during the spring 2013, as part of the Master’s degree projects by U. H. and S. S. All women scheduled for termination of pregnancy in the first trimester by vacuum aspiration or curettage, who met the inclusion criteria, were asked to participate. Inclusion criteria were healthy women assessed as American Society of Anesthesiologists physical status classes I-II, with a gestational age below 12 weeks, age above 18 years, and understanding oral and written Swedish. In all, 54 women were asked to participate, of whom 3 declined, and the recruitment stopped after 51 included patients. A sample size based on power calculation could not be performed, because in comparison with placebo, the oxytocin effects on DPA variables were unknown.

**Fig. 1:**
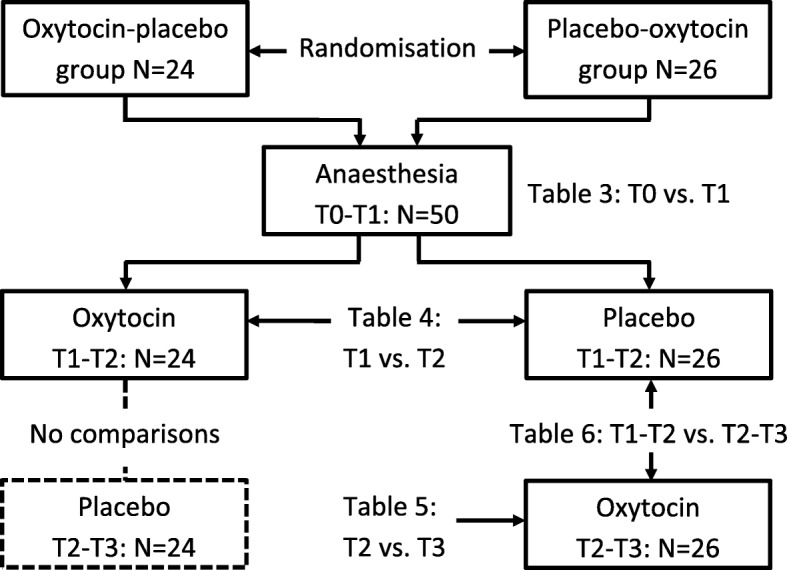
Flow chart showing the steps of the experiment. The first drugs were administered between the T1 and T2 measurement points, and the second drugs between T2 and T3

The women were randomised by P.O. using a web-based random number generator [11] to first treatment with either intravenous (IV) oxytocin or placebo. Before the start of anaesthesia, the nurse anaesthetist opened a sealed opaque envelope containing the treatment allocation. The nature of the injection was blinded to the woman, to the researchers, and to the surgeon. Women given oxytocin as the first injection were given placebo as the second injection (oxytocin-placebo sequence, OP group) and vice versa (placebo-oxytocin sequence, PO group) (Fig. 1). The oxytocin injection comprised 1 mL of Oxytocin Pilum 8.3 μg/mL (5 U) (Orifarm Generics, Stockholm, Sweden) and the placebo injection comprised 1 mL of NaCl 9 mg/mL.

The physiological background to the DPA method has been described previously [6, 8]. The Meridian DPA™ reports 17 different variables, but for this study we selected those variables with the best repeatability and best correlation to gold standard applanation tonometry: pulse height (PH), ageing index (AI), cardiac LV ejection time (LVET) compensated (ETc), cardiac ejection elasticity index (EEI), dicrotic index (DI), and the ratios *b*/*a* and *d*/*a* [5]. The variables are described in Table 1.

**Table 1:** Description of the digital pulse wave analysis variables used in the study, revised from von Wowern et al. [5]

Variable	Physiological background	Conditions with high values	Conditions with low values	Interpretation of increase	Interpretation of decrease
Pulse height (PH)	Circulation in small finger arteries, perfusion of finger tips	High BP, hyperthyroidism, fever, anemia, excessive blood volume, exercise, well-tuned athlete	Peripheral vaso-constriction, low BP, hypovolemia/dehydration, hypothyroidism, increased peripheral resistance	Peripheral vasodilatation	Peripheral vasoconstriction
Left ventricular ejection time compensated (ETc)	Represents systole, i.e. time from onset of the systolic upstroke limb to the closure of the aortic valve	Aortic valve stenosis, increased large artery compliance (low vascular tone)^a^	LV failure, decreased preload, hypovolemia, decreased large artery compliance (high vascular tone) ^a^	Increase in LV ejection time, Decreased afterload, decreased SVR, increased preload ^a^	Decrease in LV ejection time, Increased afterload, increased SVR, decreased preload ^a^
Cardiac ejection elasticity index (EEI)	Index for LV ejection capacity and compliance/elasticity of large arteries	Large artery vasodilatation, anemia, increased LV ejection power, hyperthyroidism, congested heart failure	Large artery vasoconstriction, arteriosclerosis, LV ejection insufficiency	Increase in LV ejection power, large artery vasodilatation	Decrease in LV ejection power, large artery vasoconstriction
Dicrotic index (DI)	Represents the peripheral circulation, indicates peripheral resistance	Small artery vasoconstriction	Small artery vasodilatation	Peripheral vasoconstriction	Peripheral vasodilatation
*b*/*a*	Early systolic PW peaks identified by second derivatives of the crude PW curve contour; indicates LV ejection capacity and large artery compliance/elasticity	Low large artery elasticity, increased cardiovascular risk, vasoconstriction, atherosclerosis, increases by age	Young persons, athletes	Large artery vasoconstriction, decreased LV ejection	Large artery vasodilatation, increased LV ejection
*d*/*a*	*d* is a late systolic PW peak identified by second derivative of the crude PW curve contour; mainly reflects the intensity of the tidal PW from small peripheral arteries	High small artery elasticity, young persons	A longer negative *d* peak develops by advancing age, indicating arterial stiffness, atherosclerosis	Small artery vasodilatation	Small artery vasoconstriction
Ageing index (AI)	AI = (*b*-*c*-*d*-*e*)/*a*, representing the global vascular stiffness, i.e. “vascular age”	Atherosclerosis, increases by age	Young persons, athletes	Global arterial vasoconstriction	Global arterial vasodilatation

*BP* blood pressure, *SVR* systemic vascular resistance, *LV* left heart ventricle, *PW* pulse wave

^a^) See Discussion for interpretation

**Fig. 2:**
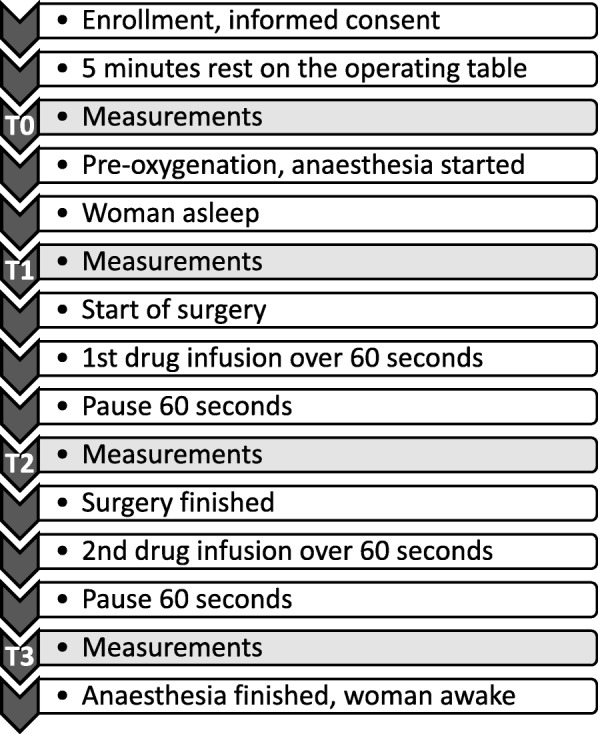
Study protocol with time sequences of monitoring, anaesthesia and intravenous drug injections

The sequences of monitoring and treatments are shown in Fig. 2. Three hours prior to scheduled surgery, the women were pre-medicated with two intravaginal misoprostol 0.2 mg tablets (Cytotec®, Pfizer AB, Sollentuna, Sweden) and two oral paracetamol 500 mg tablets (Alvedon®, GlaxoSmithKline Consumer Healthcare, Bröndby, Denmark). Upon arrival at the operating room, a peripheral IV catheter was introduced. A 5-lead ECG, a blood pressure (BP) cuff and a peripheral oxygen saturation (SaO_2_) probe were connected to the surveillance monitor (DASH 4000, GE Medical Systems Information Technologies, Danderyd, Sweden). The ECG ST index was automatically derived from the ECG lead II. Readings of BP, SaO_2_, and ST index were manually noted in a case report form.

For the DPA measurements, a customized pulse oximetry probe was placed on the right second or third finger, and then connected to the Meridian DPA™ (Meridian Co. Ltd., Korea, and Salcor AB, Uppsala, Sweden). The Meridian DPA was connected to a laptop (HP 625, Hewlett Packard, Solna, Sweden). Each DPA measurement takes 70 s to perform, during which surgery was halted.

Since the surgery was performed with the woman in the lithotomy position and the DPA measurements are sensitive to body position, the woman was placed in this position on the operating table already before initialising anaesthesia. After 5 min of rest in this position, the baseline measurements (time point T0) were performed (Fig. 2). The woman was then pre-oxygenated with 100% oxygen through a breathing mask. General anaesthesia was commenced with an injection of fentanyl 100 μg (Fentanyl® B. Braun 50 μg/mL) followed by a bolus dose of propofol (Propofol®-Lipuro B. Braun 10 mg/mL), individually titrated to loss of consciousness and eyelid reflex, after which anaesthesia was maintained with propofol only. The women were kept on mask airway during anaesthesia.

At each time point, the measurements were started with a DPA recording (lasting 70 s) paralleled by the ECG ST index reading, followed by a BP measurement. The second measurements were performed after the induction of anaesthesia, but before the start of surgery (time point T1, see Fig. 2). The first drug injection was given when the cervical dilatation was completed and uterine evacuation began. The injection time was one minute. A stopwatch was started and the point T2 DPA recording started 60 s after finishing the injection.

The second drug injection was given at the end of surgery but before end of anaesthesia and the point T3 measurements were performed in the same way as for the T2 measurements (Fig. 2).

Since the plasma half-time elimination of oxytocin is 3–20 min [12], the comparison of oxytocin vs. placebo could not be performed in the OP group after the second injection due to a too short wash-out time of oxytocin after the first injection. Thus, the oxytocin effect was in comparison with placebo studied cross-sectionally in the OP group at T1-T2 and longitudinally in the PO group for T1-T2 vs. T2-T3 (Fig. 1). For each studied variable, the difference from before until after administration was calculated, denoted Δ value. A positive Δ value denotes an increase and a negative value a decrease.

The DPA data were automatically exported to an Excel file in the laptop and later converted to a statistics software document (StatView version 5.0.1, SAS Institute, Cary, NC, USA). The Meridian manufacturer recommends that at least 80% of the pulse waves should be recorded to ensure good quality of the mathematical analyses of the pulse wave contour, so recordings with < 80% recognition were excluded from statistical analyses.

The Mann-Whitney U test was used to compare continuous variables between groups. For longitudinal comparisons within groups the Wilcoxon signed-ranks matched-pairs test was used. Categorical data were compared with Chi-2 test or Fisher’s exact probability test. Fisher’s exact test in 2 × 3 tables was performed with software available on the web [13]. Two-sided *P* values < 0.05 were considered statistically significant.

Some of the DPA variables are heart rate (HR) dependent [5] and the statistical analyses were accordingly performed with both crude and HR-adjusted DPA values. If simple linear regression analyses yielded a statistically significant correlation (*P* < 0.05) between HR and a DPA variables at T0, and the intervention (anaesthesia, oxytocin or placebo administration) also resulted in a significant change in HR, the DPA variables in question was adjusted to a HR of 75 bpm, denoted DPA@75, with the equation DPA@75 = DPA ± *C*(75-HR). *C* denotes the slope constant. The DPA variable ETc and the ECG variable ST index are automatically adjusted for HR when reported by the respective apparatus.

## Results

One patient in the OP group did not receive the allocated intervention due to a surgical complication with perforation of the uterus. Thus, 24 women allocated to the OP group and 26 to the PO group were included in the study. Their demographic characteristics are shown in Table 2.

**Table 2:**
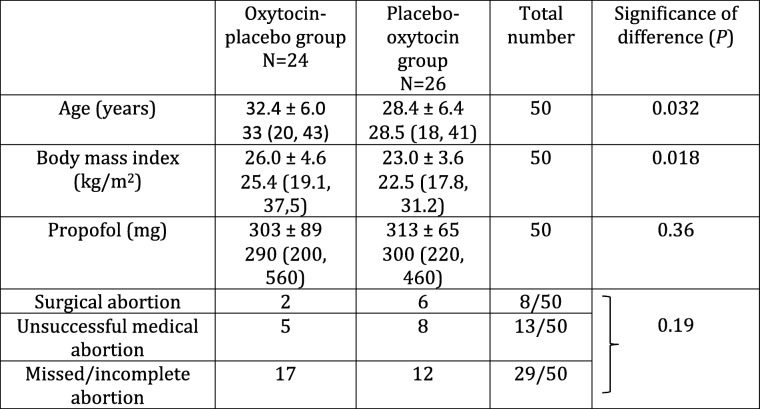
Demographic characteristics

Figures are mean ± standard deviation and median (range)

Statistics were performed with the Mann-Whitney *U* test or Fisher’s exact probability test

At time point T0, in 17/50 (34%) women the PH was below the 80% threshold for receiving adequate signals for pulse wave contour analysis, thus disabling reliable calculations of DPA variables. This excluded the longitudinal comparisons T0-T1 (effects of anaesthesia) in 34–46% of cases. For recordings T1-T3, the recording error rate was 4–8%.

At point T0, a significant relationship was found between HR and DI (*P* < 0.0001, r^2^ 0.67) and EEI (*P* = 0.017, r^2^ 0.17) and these variables were accordingly adjusted in the statistical analyses (called DI@75 and EEI@75). AI, *b*/*a* and *d*/*a* were not significantly related to HR (*P* ≥ 0.50).

Anaesthesia caused a decrease in MAP and HR and a small-artery and peripheral vasodilation, as indicated by PH and DI@75, and a global vasoconstriction was indicated by AI (Table 3).

**Table 3:** Haemodynamic effects of general anaesthesia in first trimester pregnant women. For explanation of measurement points T0 and T1, see text and Fig. 2

Variable	Number of cases	Cardiovascular effect of anaesthesia measured T0-T1			
		T0 value Mean ± SD Median (range)	T1 value Mean ± SD Median (range)	Significance of difference (*P*)	Cardiovascular effect
MAP (mmHg)	49	85 ± 12 84 (65, 112)	65 ± 9 64 (49, 89)	0.0001	Blood pressure drop
HR (bpm)	33	73 ± 14 70 (44, 96)	65 ± 9 63 (48, 84)	0.002	Heart rate drop
ECG ST index	48	0.15 ± 0.28 0.20 (− 0.70, 0.90)	0.20 ± 0.22 0.20 (− 0.30, 0.60)	0.13	
PH	33	2.0 ± 1.9 1.3 (0.6, 10.5)	5.3 ± 2.9 4.9 (0.8, 14.9)	0.0001	Peripheral vasodilation
ETc (ms)	33	374 ± 30 372 (313, 442)	361 ± 45 368 (165, 437)	0.56	
EEI	27	0.69 ± 0.28 0.59 (0.27, 1.32)	0.60 ± 0.18 0.58 (0.18, 1.00)	0.19	
EEI@75	27	0.71 ± 0.26 0.74 (0.19, 1.21)	0.68 ± 0.19 0.69 (0.28, 1.14)	0.61	
DI	33	0.66 ± 0.14 0.65 (0.37, 0.93)	0.66 ± 0.14 0.66 (0.36, 0.94)	0.31	
DI@75	33	0.65 ± 0.08 0.65 (0.50, 0.83)	0.56 ± 0.14 0.58 (0.25, 0.80)	0.0009	Small-artery vasodilation
*b*/*a*	33	−0.63 ± 0.14 − 0.64 (−1.05, − 0.39)	− 0.60 ± 0.15 − 0.56 (− 1.02, − 0.26)	0.46	
*d*/*a*	33	− 0.20 ± 0.10 − 0.17 (− 0.49, − 0.07)	− 0.18 ± 0.14 − 0.15 (− 0.54, − 0.01)	0.24	
AI	33	− 0.48 ± 0.30 − 0.53 (− 1.03, 0.24)	−0.32 ± 0.28 − 0.38 (− 0.77, 0.42)	0.010	Global vasoconstriction

*MAP*, mean arterial blood pressure, *HR* heart rate *ECG*, electrocardiogram, *PH* pulse height, *ETc* cardiac left ventricular ejection time compensated, *EEI*, cardiac ejection elasticity index, *EEI@75*, EEI adjusted to a HR of 75 bpm, *DI* dicrotic index; DI@75, DI adjusted to a HR of 75 bpm; *b*/*a*, ratio between second derivatives of crude pulse wave contour; *d*/*a*, ratio between second derivatives of crude pulse wave contour; AI, ageing index

Statistics performed with Wilcoxon matched-pairs signed-rank test

**Fig. 3:**
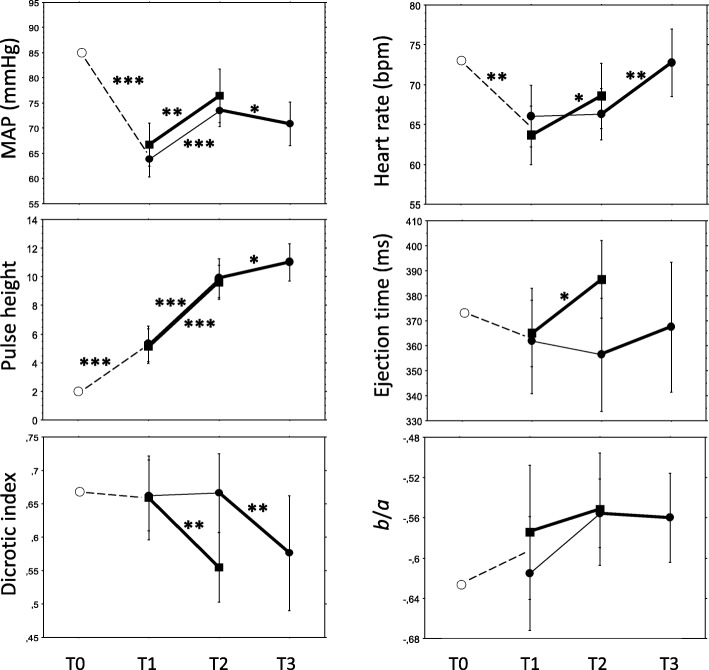
Sequential changes of mean arterial blood pressure (MAP), heart rate, pulse height, left ventricular ejection time, dicrotic index and *b*/*a* from time point T0 through T3 (for details, see text). Dashed lines represent the effect of general anaesthesia. Oxytocin/placebo were administered between T1 and T2 and vice versa between T2 and T3, where filled squares represent the oxytocin-placebo group and filled circles the placebo-oxytocin group. The oxytocin-placebo group were not analysed at T2-T3, as explained in the text. Bold lines represent oxytocin effects and thin lines placebo effects. Values are mean with 95% confidence interval. The asterisk * denotes a *P* value < 0.05, ** denotes *P* < 0.01 and *** *P* < 0.001, calculated with the Wilcoxon signed-ranks matched-pairs test.

The surgery started after point T1 (Fig. 2), and oxytocin and placebo showed from T1 to T2 the following effects (Table 4, Fig. 3):

- MAP increased by equal magnitudes in the OP and PO groups, i.e. no matter the sort of injection.
- An increase in HR was seen only after oxytocin, and the difference to placebo was confirmed by comparison of ΔHR values.
- The ECG ST index changed only after placebo, confirmed by a difference in Δ values.
- PH increased (peripheral vasodilation) by equal magnitudes after oxytocin and placebo, confirmed by the absence of difference in ΔPH values.
- ETc increased (increase in LVET, increased preload, and/or decreased afterload) only after oxytocin, but the difference in ΔETc values did not reach significance (*P* = 0.069).
- EEI and EEI@75 were not affected by oxytocin or placebo.
- Changes of DI and DI@75 after oxytocin indicated small-artery vasodilation, and the difference was confirmed by a difference in ΔDI values.
- *b*/*a*, *d*/*a* and AI did not change significantly in any group.

**Table 4:** Haemodynamic effects of 5 U (8.3 μg) oxytocin and placebo (NaCl) given intravenously to first trimester pregnant women undergoing surgical evacuation of the uterus. For explanation of time points T1 and T2, see text and Figs. 1 and 2

Variable	Effect of drug injection measured T1-T2								
	Oxytocin (OP group) N = 24				Placebo (PO group) *N* = 26				Δ value^a^ *P*
	T1 value Mean ± SD Median (range)	T2 value Mean ± SD Median (range)	*P* ^b^	Effect	T1 value Mean ± SD Median (range)	T2 value Mean ± SD Median (range)	*P* ^b^	Effect	
MAP (mmHg)	67 ± 10 65 (49, 89)	76 ± 12 76 (57, 97)	0.001	BP increase	64 ± 9 62 (52, 88)	73 ± 8 74 (57, 87)	0.0001	BP increase	0.61
HR (bpm)	64 ± 8 63 (48, 82)	68 ± 9 71 (53, 84)	0.011	HR increase	66 ± 9 65 (51, 84)	66 ± 8 65 (53, 82)	0.90	No change	0.020
ECG ST index	0.21 ± 0.24 0.30(−0.20, 0.60)	0.15 ± 0.19 0.10 (− 0.40, 0.60)	0.30	No change	0.19 ± 0.21 0.10 (− 0.30, 0.60)	0.33 ± 0.21 0.30 (0.00, 0.80)	0.0005	ST increase	0.0008
PH	5.3 ± 2.9 4.8 (0.8, 13.3)	9.6 ± 2.5 8.8 (6.1, 15.0)	< 0.0001	Peripheral vasodilation	5.3 ± 3.0 4.9 (1.8, 14.9)	9.9 ± 3.3 9.5 (4.9, 16,0)	< 0.0001	Peripheral vasodilation	0.45
ETc (ms)	360 ± 39 366 (245, 418)	381 ± 41 383 (267, 444)	0.021	Decreased afterload, decreased SVR, increased preload	362 ± 51 369 (165, 437)	356 ± 55 371 (156, 404)	0.70	No change	0.069
EEI	0.58 ± 0.19 0.58 (0.18, 0.93)	0.59 ± 0.16 0.60 (0.26, 0.77)	0.65	No change	0.61 ± 0.18 0.60 (0.31, 1.00)	0.53 ± 0.13 0.51 (0.36, 0.83)	0.12	No change	0.17
EEI@75	0.67 ± 0.18 0.66 (0.28, 0.98)	0.65 ± 0.16 0.67 (0.32, 0.95)	0.95	No change	0.69 ± 0.20 0.72 (0.32, 1.14)	0.61 ± 0.15 0.57 (0.41, 0.96)	0.23	No change	0.43
DI	0.65 ± 0.15 0.67 (0.36, 0.94)	0.55 ± 0.11 0.55 (0.33, 0.77)	0.001	Small-artery vasodilation	0.66 ± 0.13 0.66 (0.41, 0.92)	0.67 ± 0.14 0.66 (0.41, 0.99)	0.84	No change	0.015
DI@75	0.55 ± 0.14 0.57 (0.25, 0.77)	0.49 ± 0.11 0.50 (0.32, 0.65)	0.023	Small-artery vasodilation	0.58 ± 0.14 0.58 (0.28, 0.80)	0.59 ± 0.15 0.61 (0.22, 0.88)	0.97	No change	0.10
*b*/*a*	− 0.59 ± 0.16 − 0.54 (− 0.95, − 0.26)	−0.57 ± 0.13 − 0.57 (− 0.85, − 0.25)	0.74	No change	−0.62 ± 0.14 − 0.61 (− 1.02, − 0.40)	−0.56 ± 0.08 − 0.55 (− 0.78, − 0.39)	0.094	No change	0.19
*d*/*a*	−0.17 ± 0.14 − 0.14 (− 0.54, − 0.01)	− 0.20 ± 0.09 − 0.20 (− 0.39, − 0.08)	0.38	No change	−0.18 ± 0.14 − 0.18 (− 0.50, − 0.01)	− 0.23 ± 0.13 − 0.20 (− 0.76, − 0.08)	0.084	No change	0.84
AI	− 0.31 ± 0.29 − 0.38 (− 0.77, 0.21)	−0.27 ± 0.24 − 0.27 (− 0.63, 0.24)	0.99	No change	−0.32 ± 0.29 − 0.37 (− 0.70, 0.42)	−0.25 ± 0.20 − 0.30 (− 0.50, 0.25)	0.086	No change	0.27

*OP* group, women given oxytocin at first injection and placebo at second injection; PO group, women given placebo at first injection and oxytocin at second injection

^a)^ The Δ values are not shown, but represent the T2 value minus T1 value. *P* denotes significance of difference in Δ value between oxytocin and placebo injections, performed with the Mann-Whitney *U* test

^b)^ *P* denotes significance of difference between T1 and T2 values, performed with the Wilcoxon matched-pairs signed-ranks test

Due to a probable insufficient wash-out effect of oxytocin given as the first injection in the OP group, the effects of placebo given as the second injection T2-T3 were not analysed (Fig. 1). Table 5 shows the effects of oxytocin when comparing T2 and T3 values. Effects were found on MAP, HR, ST index, PH, DI and DI@75, whereas no effects were found on ETc, EEI, EEI@75, *d*/*a* and AI. A decrease in *b*/*a* did not reach statistical significance (*P* = 0.056, which would indicate large-artery vasodilation and increase in LV ejection if significant).

**Table 5:** Haemodynamic effects of 5 U (8.3 μg) oxytocin given as the second injection at T2-T3 in women given placebo as the first injection

Variable	Oxytocin (PO group) *N* = 26			
	T2 value Mean ± SD Median (range)	T3 value Mean ± SD Median (range)	*P* ^a^	Effect
MAP (mmHg)	73 ± 8 74 (57, 87)	71 ± 11 71 (51, 90)	0.046	BP decrease
HR (bpm)	66 ± 8 65 (53, 82)	73 ± 10 72 (53, 88)	0.001	HR increase
ECG ST index	0.33 ± 0.21 0.30 (0.00, 0.80)	0.18 ± 0.29 0.20 (− 0.40, 0.70)	0.014	ST decrease
PH	9.9 ± 3.3 9.5 (4.9, 16,0)	11.0 ± 3.0 11.5 (5.5, 16.0)	0.019	Peripheral vasodilation
ETc (ms)	356 ± 55 371 (156, 404)	367 ± 60 381 (179, 430)	0.39	No change
EEI	0.53 ± 0.13 0.51 (0.36, 0.83)	0.50 ± 0.13 0.48 (0.33, 0.80)	0.33	No change
EEI@75	0.61 ± 0.15 0.57 (0.41, 0.96)	0.52 ± 0.15 0.52 (0.31, 0.84)	0.33	No change
DI	0.67 ± 0.14 0.66 (0.41, 0.99)	0.58 ± 0.20 0.53 (0.26, 0.99)	0.006	Small-artery vasodilation
DI@75	0.59 ± 0.15 0.61 (0.22, 0.88)	0.56 ± 0.20 0.55 (0.12, 1.06)	0.013	Small-artery vasodilation
*b*/*a*	−0.56 ± 0.08 − 0.55 (− 0.78, − 0.39)	− 0.56 ± 0.10 − 0.57 (− 0.74, − 0.35)	0.056	No change
*d*/*a*	−0.23 ± 0.13 − 0.20 (− 0.76, − 0.08)	− 0.22 ± 0.09 − 0.21 (− 0.40, − 0.04)	0.42	No change
AI	−0.25 ± 0.20 − 0.30 (− 0.50, 0.25)	−0.20 ± 0.19 − 0.20 (− 0.68, 0.05)	0.15	No change

^a)^ *P* denotes significance of difference between T2 and T3, performed with the Wilcoxon matched-pairs signed-ranks test

At point T3 surgery had finished, but the women were still under anaesthesia (Fig. 2). In longitudinal comparisons in the PO group, the effects of oxytocin measured T2-T3 were compared with the effects of placebo measured T1-T2 (Table 6, Fig. 1). EEI and EEI@75 statistics could not be performed due to too few cases in comparisons (*N* = 10). The results in Table 6 are interpreted in Table 7, together with an overall interpretation of oxytocin effects displayed in Tables 4-6.

**Table 6:** Haemodynamic effects of oxytocin (administered during sequence T2-T3) relative to placebo (sequence T1-T2) in first trimester pregnant women in the placebo-oxytocin group (*N* = 26). For interpretation and comparisons of oxytocin effects, see Table 7

Variable	Placebo Δ value T1-T2^a^ Mean ± SD Median (range)	Oxytocin Δ value T2-T3^b^ Mean ± SD Median (range)	Significance of difference^c^ (*P*)
MAP (mmHg)	10 ± 9 10 (− 13, 23)	− 3 ± 7 − 2 (− 16, 12)	0.0002
HR (bpm)	0 ± 5 0 (− 13, 9)	6 ± 7 4 (− 4, 22)	0.020
ECG ST index	0.14 ± 0.16 0.10 (− 0.10, 0.50)	− 0.15 ± 0.28 − 0.10 (− 0.90, 0.30)	0.001
PH	4.7 ± 2.9 4.1 (− 0.8, 10.1)	1.0 ± 1.8 0.9 (− 2.8, 3.4)	0.005
ETc (ms)	−3 ± 48 5 (− 200, 55)	2 ± 48 6 (− 160, 107)	0.70
DI	0.00 ± 0.18 − 0.02 (− 0.41, 0.46)	− 0.11 ± 0.16 − 0.14 (− 0.34, 0.30)	0.016
DI@75	0.00 ± 0.18 0.00 (− 0.39, 0.49)	−0.06 ± 0.17 − 0.01 (− 0.25, 0.50)	0.11
*b*/*a*	0.06 ± 0.15 0.02 (− 0.19, 0.53)	−0.02 ± 0.08 − 0.05 (− 0.14, 0.21)	0.024
*d*/*a*	−0.04 ± 0.13 − 0.03 (− 0.39, 0.27)	−0.02 ± 0.09 − 0.00 (− 0.18, 0.16)	0.78
AI	0.07 ± 0.23 0.08 (− 0.55, 0.62)	0.06 ± 0.20 0.04 (− 0.26. 0.47)	0.94

^a)^ Δ value calculations *T2* value minus T1 value

^b)^ Δ value calculations: T3 value minus T2 value

^c)^ Statistics performed with Wilcoxon matched-pairs signed-ranks test, comparing the changes (Δ value) obtained by oxytocin and placebo

**Table 7:** Summary of observations based on haemodynamic effects of oxytocin administered at first injection T1-T2 (Table 4) and second injection T2-T3 (Tables 5 and 6). For further explanation of comparisons, see Fig. 1, and for explanation of interpretations, see text

Variable	Table 4 T1-T2 in OP group *N* = 24	Table 5 T2-T3 in PO group *N* = 26	Table 6 T1-T2 vs. T2-T3 in PO group *N* = 26	Summary of observations
MAP (mmHg)	No change^a^	Decrease	Decrease	Decrease
HR (bpm)	Increase	Increase	Increase	Increase
ECG ST index	No change	Decrease	Decrease	Decrease
PH	No change^b^	Increase ➔ peripheral vasodilation	Increase ➔peripheral vasodilation	Increase ➔ peripheral vasodilation
ETc (ms)	Increase ➔ large-artery vasodilation, decreased afterload, decreased SVR, increased preload	No change	No change	Increase ➔ large-artery vasodilation, decreased afterload, decreased SVR, increased preload
EEI	No change	No change	N/A^c^	No change
EEI@75	No change	No change	N/A^c^	No change
DI	Decrease ➔ small-artery vasodilation	Decrease ➔ small-artery vasodilation	Decrease ➔ small-artery vasodilation	Decrease ➔ small artery vasodilation
DI@75	Decrease ➔ small-artery vasodilation	Decrease ➔ small-artery vasodilation	No change	Decrease ➔ small artery vasodilation
*b*/*a*	No change	No change	No change/decrease ➔ large-artery vasodilation, increase in LV ejection	No change
*d*/*a*	No change	No change	No change	No change
AI	No change	No change	No change	No change

^a)^ *MAP* increased after both oxytocin and placebo

^b)^ *PH* increased after both oxytocin and placebo, indicating peripheral vasodilation

^c)^ *EEI* and *EEI*@75 statistics could not be performed due to too few cases in comparisons (N = 10)

Figure 3 shows the sequential variable changes throughout the experiment from T0 to T3, and Table 7 shows a comprehensive interpretation of oxytocin effects, where some interpretations need further explanations. The MAP increased at T1-T2 in both the oxytocin and placebo groups with no significant difference in magnitude (Table 4), whereas the difference to placebo in the comparison T1-T2 vs. T2-T3 was significant (Table 6); as oxytocin at T2-T3 caused a decrease (Tables 5 and 6) and placebo at T1-T2 an increase (Table 4), the comprehensive interpretation is that MAP decreased after oxytocin (Table 7).

The ECG ST index was unchanged after oxytocin, whereas it increased after placebo at T1-T2 comparisons (Table 4). The difference to placebo in the comparison T1-T2 vs. T2-T3 was significant, where oxytocin showed a decrease and placebo an increase (Tables 4-6). The interpretation is that ECG ST index decreased after oxytocin (Table 7).

The ETc increased at T1-T2 after oxytocin whereas placebo had no effect (Table 4). In the longitudinal comparison with placebo at T1-T2 vs. T2-T3 (Table 6) there was no difference to placebo, and at T2-T3 oxytocin had no effect on ETc (Table 5). The findings were then not unanimous.

No harmful side effects were noted during the study and no woman complained of chest discomfort.

## Discussion

This study supports the hypothesis that oxytocin decreases vascular tone. The changes in DPA variables after an oxytocin bolus suggest vasodilation and decreased vascular tone of small and peripheral arteries. This was established at both cross-sectional and longitudinal comparisons with placebo. The PH, an indicator of the peripheral circulation, increased after anaesthesia and then further after the first drug injection, of equal magnitude after oxytocin and placebo. PH has previously been found to increase with increasing depth of anaesthesia due to reduced sympathetic activity [14]. Then, a continuously increasing PH with increasing depth of anaesthesia might explain the placebo effect of the first injection.

The second injection was given when surgery was finished and the depth of anaesthesia was at steady state or ceasing. Then, oxytocin caused small-artery and peripheral vasodilation, fall of MAP and increase in HR, which further supports the conclusion that oxytocin causes a small-artery and peripheral vasodilation.

The increase in MAP in both groups at the start of surgery might be the result of an increase in sympathetic activity due to painful stimuli. It is, however, possible that a fall in MAP of a very short duration failed to be captured in our study protocol, as MAP was measured after the one-minute DPA recording was completed. The BP measurement takes up to 30 s, meaning that MAP was measured 2–3 min after the end of the oxytocin injection. It has been demonstrated in previous studies that the MAP reduction reaches its nadir already within 1–2 min, and returns to normal after another minute [15, 16]. The ECG changes also occur within 1–2 min [17]. Furthermore, oxytocin has a biphasic effect, with a rapid initial HR increase and decrease in systolic BP followed by a fall in HR and increase in BP [3, 18]. We might then not have captured the rapid initial effect of oxytocin in our study. Also the duration of the bolus injection has been attributed to the nadir lag time. Some researchers used *statim* bolus and others, like us, used a 60 s injection time. A short injection time might maximise the initial effects. Since the BP measurements could interfere with the DPA measurements, we had no choice but to await finishing the DPA measurement before we could record the BP.

The ETc increased after oxytocin at T1-T2 whereas placebo had no effect. Considering the whole experiment, the effect of oxytocin on ETc was not unanimous, but as the T1-T2 comparison was the only one performed during equal conditions in the OP and PO groups, our overall interpretation is that oxytocin caused an increase in ETc. According to our previous communications with the South Korean manufacturer of the Meridian DPA, an increase in ETc could mean aortic valve stenosis or LV failure, as previously described [4, 5]. It might be true concerning aortic valve disease, but is more complex concerning LV failure [19]. Both ETc and the similar measure Flow Time Corrected, derived from oesophageal Doppler velocimetry of aortic blood flow, correlate strongly with the LVET [5, 20]. Lewis et al. pointed out already in 1977 that LV muscle failure and diminished preload is associated with a shortened LVET, and that a marked increase in SV would lengthen the LVET [19].

Later on, Singer et al. [21, 22] using oesophageal Doppler velocimetry, demonstrated that the LVET was increased when preload was increased from a hypovolemic state, but also when preload was decreased from an overload state. Thus, LVET is reaching its maximum at optimal filling pressures. As preload diminishes, compensatory peripheral vasoconstriction causing increased systemic vascular resistance (SVR) usually occur. In effect, vasodilating agents alone also produce an increase in LVET, leaving preload unaffected. Taken together, in healthy patients without fluid overload, LVET increases not only by increased preload but also by decreased afterload, i.e. from decreased SVR [23].

From this argumentation, we believe that the rise in ETc after the first oxytocin injection indicates a reduction in SVR, since preload is assumed unaffected. As HR increased without a fall in MAP, an increase in CO is assumed. An increase in CO leads to an increase in myocardial oxygen demand. Signs of myocardial ischemia have previously been noted after oxytocin injection [17], and that observation could explain the different responses in ECG ST index after oxytocin and placebo.

Thirty-four percent of DPA values were missing at T0 due to cold fingers, probably caused by pre-operative stress-induced peripheral vasoconstriction [14]. Among confounding factors, sympathetic activity induced by noxious stimuli from the surgical procedure might explain changes seen in the placebo group between T1 and T2. Although there were no differences between the groups in the total doses of propofol given, variations in anaesthetic depth is another possible confounding factor.

The cardiovascular effects of anaesthesia included decreases in BP and HR and dilatation of small and peripheral arteries. However, the global index AI indicated vasoconstriction. AI is a composite index of four indices reflecting different phases of the pulse wave from early systole to early diastole, i.e. *b*/*a*, *c*/*a*, *d*/*a* and *e*/*a* [5]. The two indices reported separately in the present study, i.e. *b*/*a* and *d*/*a*, did not change significantly, and the variable reflecting large arteries, EEI, did not change either. Then, the significant change of AI can be interpreted as the amalgamated result of non-significant changes of the subset of indices, or it was a result of a type 1 error. Oxytocin has both vasodilatory and vasoconstrictory properties via the same receptor, V1aR [24], and various vascular beds and organs display different distributions and receptor sensitivity [25]. Although not possible to demonstrate in the present study, vasoconstriction in medium-sized arteries could be a physiological response to peripheral vasodilation and falls in BP and HR.

DPA with digital photoplethysmography is a non-invasive, operator-friendly and rapid method to study vascular tone and pharmacological hemodynamic effects in a clinical setting where the effects come swift. As illustrated by the DPA measurements before initializing anaesthesia, cold fingers must be avoided as they distort the results. That applies to finger movements as well.

A strength of the study is the randomised double-blinded placebo-controlled design in a normal clinical setting. In comparison with our previous study on oxytocin during CS, where the procedures with spinal anaesthesia, IV fluids, vasoactive drugs and delivery of the baby made it problematic to interpret the hemodynamic effects of oxytocin [4], the present study was less compromised by confounding factors. Another strength is the DPA method’s ability to investigate effects on large and small arteries separately, by using derivative mathematics to analyse the systolic and diastolic segments of the pulse wave contour. It is a limitation that the study protocol could not be adapted to capture short biphasic courses of events. As surgery needed to be stopped during DPA and BP measurements, for the safety of the women we could not perform double measurements, as would have been experimentally preferred. Variations in anaesthetic depth and sympathetic activity are possible confounders that could not be controlled for.

Misoprostol 0.4 mg was administered vaginally 3 h prior to scheduled surgery. The drug has a plasma half-time of about 30 min, but due to slow absorption by the vaginal route the bioactivity could remain for a few hours [26]. It is therefore possible that remainders of misoprostol persisted at the time of the experiments. However, the lasting bioactivity has been shown on the myometrium [26], whereas no cardiovascular effects of misoprostol have been demonstrated [27, 28]. We therefore believe possible remainders of misoprostol did not confound the results. The placebo-controlled design of the study could ensure it was not misoprostol effects that we measured, and the longitudinal measurements were performed during a short time-span when steady-state concentrations of misoprostol would not bias the results.

## Conclusions

This study confirmed the hypothesis that oxytocin decreases vascular tone in small and peripheral arteries, resulting in a lower BP and a compensatory increase in HR. These effects might have been enhanced by the vasodilating effects of anaesthesia. We could not confirm any effect on vascular tone in large arteries. The net oxytocin effect on the heart is likely to be an increased CO, implying increased myocardial oxygen demand. The demonstrated multiple effects of oxytocin on the cardiovascular system suggest a cautious use of oxytocin in pregnant women.

## Abbreviations

AI: Ageing index
b/a: Second derivative quotient of the acceleration phase the percussion pulse wave
BP: Blood pressure
CO: Cardiac output
CS: Caesarean section
d/a: Second derivative quotient of percussion wave clashing the reflected tidal wave
DI: Dicrotic index
DI@75: DI adjusted to a heart rate of 75 bpm
DPA: Digital pulse wave analysis
ECG: Electrocardiogram
EEI: Ejection elasticity index
EEI@75: EEI adjusted to a heart rate of 75 bpm
ETc: Left ventricular ejection time compensated (for heart rate)
Ftc: Flow time corrected
HR: Heart rate
IV: Intravenous
LV: Left ventricle of the heart
LVET: Left ventricular ejection time
MAP: Mean arterial blood pressure
OP: Oxytocin-placebo sequence
PH: Pulse height
PO: Placebo-oxytocin sequence
ST index: Difference in ECG ST segment from the isoelectric baseline
SVR: Systemic vascular resistance
Δ value: Difference between measurement at time point T_n_ minus measurement at point T_n-1_
